# GATA zinc finger protein p66β promotes breast cancer cell migration by acting as a co-activator of Snail

**DOI:** 10.1038/s41419-023-05887-w

**Published:** 2023-06-28

**Authors:** Xiuqun Zou, Li Ma, Yihong Zhang, Qun Zhang, Chu Xu, Dan Zhang, Yimin Chu, Jie Zhang, Mengying Li, Hui Zhang, Jiamin Wang, Chicheng Peng, Gang Wei, Yingjie Wu, Zhaoyuan Hou, Hao Jia

**Affiliations:** 1 Hongqiao Institute of Medicine, Tongren Hospital/Faculty of Basic Medicine, Shanghai Jiaotong University School of Medicine, Shanghai, China; 2 Shanghai Key Laboratory for Tumor Microenvironment and Inflammation, Department of Biochemistry & Molecular Cellular Biology, Shanghai Jiaotong University School of Medicine, Shanghai, China; 3 Key Laboratory of Computational Biology, CAS-MPG Partner Institute of Computational Biology, Shanghai Institute for Biological Science, Chinese Academy of Sciences, Shanghai, China; 4 Digestive Endoscopy Center, Shanghai Tongren Hospital, Shanghai Jiaotong University School of Medicine, Shanghai, China; 5 Naruiboen Biomedical Technology Corporation Limited, Linyi, Shandong China; 6 Shandong Provincial Hospital, Shandong Laboratory Animal Center, Science and Technology Innovation Center, Shandong First Medical University & Shandong Academy of Medical Sciences, Jinan, Shandong China; 7 Key Laboratory of Cell Differentiation and Apoptosis of Chinese Ministry of Education, Shanghai, China; 8 Linyi University-Shanghai Jiaotong University Joint Institute of Translational Medicine, Linyi, Shandong China

**Keywords:** Transcriptional regulatory elements, Breast cancer, Metastasis

## Abstract

The transcriptional repressor Snail induces EMT during embryonic development and tumor metastasis. Growing evidence indicates that Snail functions as a trans-activator to induce gene expression; however, the underlying mechanism remains elusive. Here, we report that Snail cooperates with GATA zinc finger protein p66β to transactivate genes in breast cancer cells. Biologically, depletion of p66β reduces cell migration and lung metastasis in BALB/c mice. Mechanistically, Snail interacts with p66β and cooperatively induces gene transcription. Notably, a group of genes induced by Snail harbor conserved G-rich cis-elements (5′-GGGAGG-3′, designated as G-box) in their proximal promoter regions. Snail directly binds to G-box via its zinc fingers and transactivates the G-box-containing promoters. p66β enhances Snail binding affinity to G-box, whereas depletion of p66β results in a decreased binding affinity of Snail to the endogenous promoters and concomitantly reduces the transcription of Snail-induced genes. Taken together, these data demonstrated that p66β is critical for Snail-mediated cell migration by acting as a co-activator of Snail to induce genes containing G-box elements in the promoters.

## Introduction

The transcription factor Snail is a prominent inducer of the epithelial–mesenchymal transition (EMT) during embryonic development and cancer progression [1–5]. In many cancer cells, elevated Snail expression induces stem cell-like phenotypes accompanied by immune escape, chemoresistance, and metastasis [6–10]. These functions are mainly attributed to Snail as a potent transcriptional repressor that initiates the EMT program, as evidenced by the downregulation of various epithelial markers such as E-cadherin and Claudins [2, 11–13].

Snail represses gene transcription by relying on the cooperation of conserved tandem zinc finger motifs located at the C-terminus and the Snail/Gfi-1 (SNAG) domain at the N-terminus. Zinc fingers specifically bind to E-box elements (5′-CANNTG-3′) residing in their target promoters, whereas the SNAG motif recruits multiple repressive complexes to silence gene transcription [14, 15]. These complexes include histone deacetylases, mSin3A, Suv39H1, LSD1, Ring1A/B, and Ajuba/Prmt5/14-3-3 ternary complexes, which are involved in histone modifications such as acetylation, methylation, and ubiquitination [16–22].

Notably, growing evidence indicates that Snail can transactivate gene expression directly, but distinct models are proposed to explain the transactivation of Snail [23]. For example, Snail binds to the E-box-rich regions of *ERCC1* and *MMP15* promoters to induce their expression by a potential mechanism that Snail interacts with CBP to acetylate K146 and K187 residues, which results in preventing the formation of the repressor complex [24–26]. The Snail homolog CES-1 in C. *elegans* induces the expression of genes containing bHLH transcription factor binding elements and E-box elements [27]. Notably, new models suggest that transcriptional activation by Snail is dependent on novel transcriptional motifs or elements rather than the canonical E-box sequence. A novel Snail-responsive element (5′-TCACA-3′) was identified in the promoters of *ZEB1*, *MMP9*, and *p15*^*INK4b*^ genes activated by Snail in collaboration with EGR-1 and SP-1 [28, 29]. p65NF-kB, PARP1, and Snail form multiple protein complexes at the *Fibronectin 1* (*FN*) promoter to induce *FN* transcription [30]. In Drosophila Snail is also shown to induce the expression of genes critical for mesoderm development by collaborating with Twist [31]. Nevertheless, the exact mechanism by which Snail induces gene expression still remains elusive.

The p66α (GATAD2A) and p66β (GATAD2B) proteins are two members of the p66 family that contain a GATA zinc finger in conservative region two (CR2) in the C-terminal [32]. Classical vertebrate GATA transcription factors have two zinc fingers and comprise the general configuration of Cys-X2-Cys-X17-Cys-X2-Cys. While the zinc finger in the C-terminal specifically recognizes the consensus sequence A/T(GATA)A/G in the target promoters, the N-terminal zinc finger predominantly interacts with partner proteins such as the FOG family proteins to stabilize the DNA-protein complex [33, 34]. However, the GATA zinc finger in p66α/p66β has not been reported to be capable of binding to DNA or protein.

The p66 family was first identified as a component of the highly conserved, ATP-dependent, and nucleosome remodeling and deacetylase (NuRD) complex that dominates a transcriptional repression process on methylated CpG islands depending on its nucleosome remodeling and deacetylation activity [35]. While the CR1 domain of p66 mediates the interaction between p66 and other NuRD components, the CR2 domain of p66 is essential for targeting the NuRD complex to specific nuclear loci and for mediating histone tail interaction [32, 36]. Thus, p66β proteins are usually reported to exert transcriptional repression function as a component of the NuRD complex to participate in various functions, such as regulating progesterone receptor and neurodevelopmental disorders [37, 38]. However, Grzeskowiak et al. reported that p66β induces Myc expression in KRAS-mutant lung cancer cells, indicating that p66β could function as a transcriptional activator [39].

In this study, we report that Snail interacts with the CR2 domain of p66β via its zinc finger. p66β and Snail transactive genes that are activated but not repressed by Snail contain a conserved G-rich cis-element (5′-GGGAGG-3′) in the proximal target promoter regions. Snail cooperates with p66β to transactivate gene transcription by binding to G-rich cis-elements within its target promoters.

## Results

### Snail interacts with p66β

**Fig. 1:**
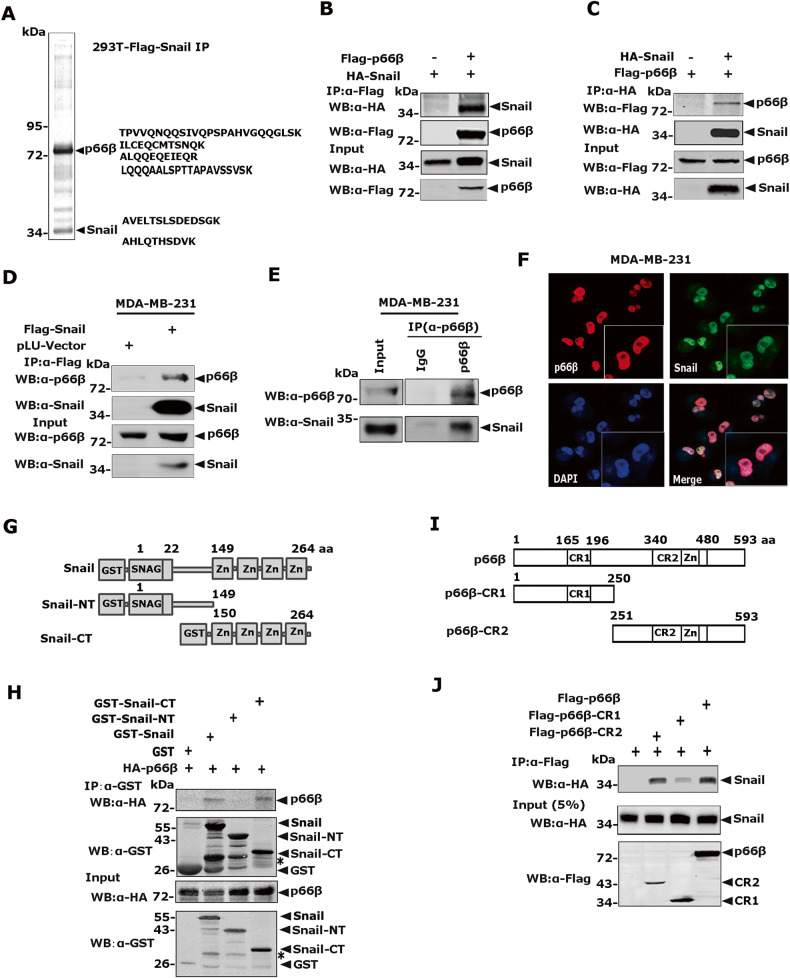
Snail binds the CR2 domain of p66β through its zinc finger motifs. **A** Colloidal staining showed that p66β was a potential Snail-interacting protein purified from HEK-293 cells. **B**, **C** Plasmids encoding Flag- p66β and HA- Snail were transiently transfected into HEK-293T cells, the cell lysates were incubated with either anti-Flag M2 beads or HA antibody and the co-eluted proteins were blotted with anti-HA (**B**) or anti-Flag (**C**) antibodies. α, anti; WB, Western blotting. **D** Snail interacted with p66β at the endogenous level. Lysates prepared from MDA-MB-231-Flag-Snail stable cells were incubated with anti-Flag M2 beads, and the co-eluted proteins were detected with an anti-p66β antibody. **E** Lysates prepared from MDA-MB-231 cells were incubated with an anti-p66β antibody and protein A/G beads, and the co-eluted proteins were detected with a Snail antibody. **F** Co-localization of Snail and p66β in MDA-MB-231 cells. Endogenous Snail and p66β in MDA-MB-231 cells were detected for immunofluorescence staining using anti-Snail and anti-p66β antibodies, and the images were taken by confocal microscopy. Scale bar: 90 μm. **G** Schematic diagrams showed full-length and truncated Snail proteins. **H** The C-terminal zinc fingers of the Snail interacted with p66β. Bacterially expressed GST-Snail and its truncation proteins, together with HA-p66β proteins prepared from HEK-293T cells were incubated with GST beads, and co-eluted proteins were analyzed by anti-HA. *non-specific band. **I** Schematic diagrams showed full-length and truncated forms of p66β. The CR2 domain contains a GATA-type zinc finger motif. **J** The CR2 domain of p66β interacted with Snail. Lysates prepared from HEK-293T cells transfected with plasmids encoding Flag-p66β and its truncation mutants, together with HA-Snail were incubated with anti-Flag M2 beads, and co-eluted proteins were analyzed by anti-HA.

To identify molecules that can cooperate with Snail to promote metastasis, we established HEK-293T cells stably expressing Flag-Snail and performed affinity purification assays. The co-eluents were resolved by SDS-PAGE followed by silver staining, and the specific band at 66 kDa was identified as p66β (Fig. 1A). To confirm the interaction between Snail and p66β, we co-expressed HA-Snail and Flag-p66β in HEK-293T cells and performed reciprocal co-IP assays. Indeed, Snail and p66β interacted with each other (Fig. 1B, C). Moreover, stably expressed Flag-Snail co-immunoprecipitated with the endogenous p66β protein (Fig. 1D). Reciprocally, p66β immunoprecipitated with Snail endogenously (Fig. 1E). To determine the subcellular localization of Snail and p66β, indirect immunofluorescence assays were performed in MDA-MB-231 cells using antibodies specifically against Snail or p66β (Fig. 1F). Notably, both Snail and p66β proteins co-localized in the nucleus.

### Snail binds the CR2 domain of p66β through its zinc finger region

To determine the regions in Snail responsible for p66β binding, full-length of GST-Snail, truncated zinc-finger structure (GST-Snail-CT), and SNAG domain (GST-Snail-NT) proteins were incubated with HA-p66β protein prepared from HEK-293T cells. The co-precipitation experiments were performed by using GST beads, and the co**-**eluted HA- p66β protein was examined by Western blotting assays using an anti-HA antibody. GST-Snail-CT and the full-length of GST-Snail showed similar binding affinities to p66β, whereas GST-Snail-NT showed no binding (Fig. 1G, H).

To identify regions in p66β that are critical for the interaction between p66β and Snail, p66β full-length or truncations were transiently co-expressed with HA**-**Snail in HEK-293T cells. Co-IP assays demonstrated that the CR2 domain displayed strong binding activity toward Snail, which was comparable to that of full-length p66β, whereas the CR1 domain showed weak binding activity (Fig. 1I, J). Taken together, these data indicated that p66β is a novel Snail-interacting protein and that its binding is mediated by the zinc finger region of Snail and the CR2 region of p66β.

### p66β promotes breast cancer cell migration and metastasis

**Fig. 2:**
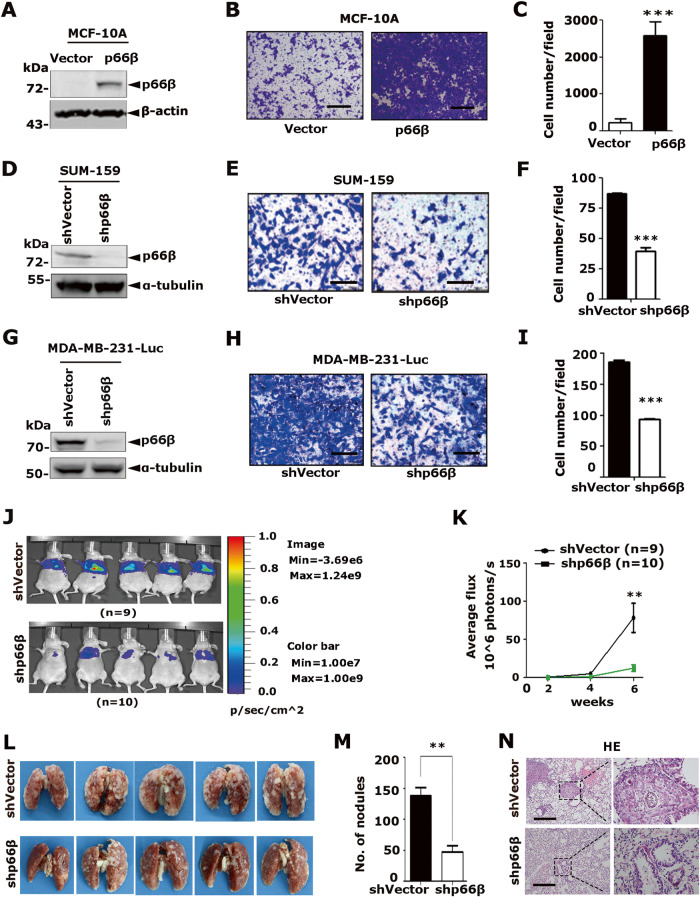
p66β promotes breast cancer cell migration and metastasis. **A**–**C** Ectopic expression of p66β induced cell migration in MCF-10A cells by transwell assay (**A**). Images represent one microscopic field in each group (**B**). Scale bar: 200 μm. Quantification of migrated cells is shown (**C**) (*n* = 5 fields were randomly chosen and counted for statistical analysis). ***p < 0.001. **D**–**F** Depletion of p66β inhibited the migration of SUM-159 cells. Scale bar: 100 μm (*n* = 9 fields were randomly chosen and counted for statistical analysis). ***p < 0.001. **G**–**I** Depletion of p66β inhibited the migration of MDA-MB-231 cells. Scale bar: 100 μm (*n* = 9 fields were randomly chosen and counted for statistical analysis). ***p < 0.001. **J**, **K** MDA-MB-231-Luc cells stably expressing p66β shRNA or shVector were injected into the tail veins of female nude mice. The development of lung metastases was recorded on the 2nd, 4th, and 6th week using luciferase bioluminescence imaging and quantified by measuring the photon flux compared with the mock group (*n* = 9 and *n* = 10 mice, respectively). **p < 0.01. **L**, **M** Depletion of p66β decreased the number of metastatic lung nodules. **p < 0.01. **N** Lung metastatic nodules were detected in paraffin-embedded sections stained with hematoxylin and eosin. Images were taken at 10x magnification with a scale bar of 200 μm; images were taken at 40× magnification with a scale bar of 50 μm). All data are shown as the mean ± S.D. by Student’s t-test.

To examine the potential function of p66β in breast cancer, we analyzed the expression of p66β, Snail, and EMT markers in breast cancer cells, and p66β was highly expressed in triple-negative cells (Fig. S1A). To determine the role of p66β in breast cancer cell migration, p66β was stably expressed in MCF-10A cells, and the resulting cells were subjected to transwell assays (Fig. 2A and Fig. S1B). Ectopic expression of p66β or CR2 truncation apparently enhanced the migratory ability of MCF-10A and MDA-MB-231 cells (Fig. 2B, C and Fig. S1E–G). Conversely, depletion of p66β resulted in a significant decrease in the migratory abilities of SUM-159 and MDA-MB-231 cells (Fig. 2D–I and Fig. S1C, D). More importantly, depletion of p66β in luciferase-labeled MDA-MB-231 cells greatly decreased lung colonization, as indicated by the lower luciferase intensity and fewer and smaller micro-metastatic nodules in BALB/c female nude mice, in which the resulting cells were injected via the tail vein (Fig. 2J–N). Collectively, these data demonstrated that p66β promotes breast cancer cell migration and metastasis.

### p66β is required for Snail-mediated cell migration

**Fig. 3:**
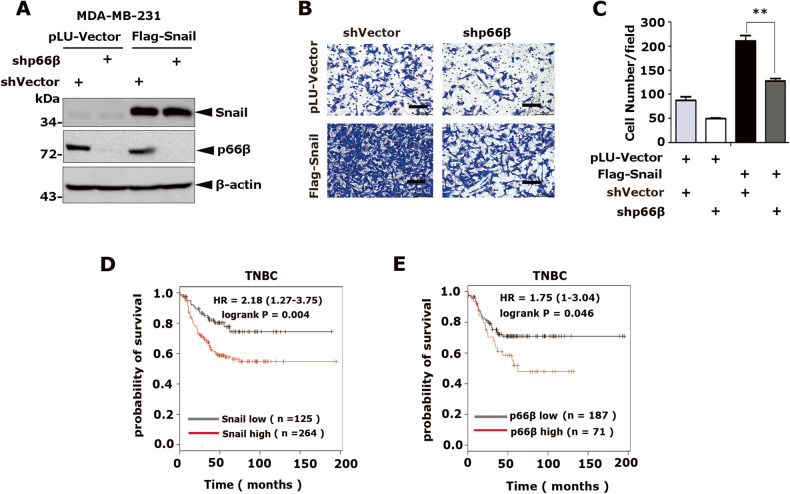
p66β is required for Snail-mediated cell migration. **A** Depletion of p66β in Snail stably expressed MDA-MB-231 cells. Western blotting showing the protein levels of Snail and p66β. **B** Knockdown of p66β reduced the cell migration mediated by Snail. Scale bar: 100 μm. **C** Statistical analysis of the migrating cells from the transwell assays was shown in the bar graphs (*n* = 9 fields were randomly chosen and counted for statistical analysis). The data are shown as the mean ± S.D. by Student’s t-test. **p < 0.01. **D**, **E** Kaplan–Meier plots of the relapse-free survival of patients with triple-negative (ER-/PR-/HER2-) breast cancer (TNBC) in the whole data set stratified by Snail and p66β expression. Data were acquired from the Kaplan–Meier plotter database. The *p*-value for Kaplan–Meier curve was determined using a log-rank test, Statistical significance was set at *p* < 0.05.

To determine whether p66β is critical for Snail-mediated cell migration and metastasis, we depleted p66β in MDA-MB-231-Snail or -Vector cells, respectively (Fig. 3A). The transwell assays showed that depletion of p66β markedly dampened Snail-mediated cell migration (Fig. 3B, C). Notably, knockdown of Snail also decreased p66β-induced cell migration ability (Fig. S2). Moreover, we analyzed the Kaplan–Meier plotter database and found that high expression of Snail and p66β was predictive of poor prognosis in patients with triple**-**negative (ER-/PR-/HER2-) breast tumors, respectively (Fig. 3D, E).

### Snail and p66β cooperatively induce target gene transcription

**Fig. 4:**
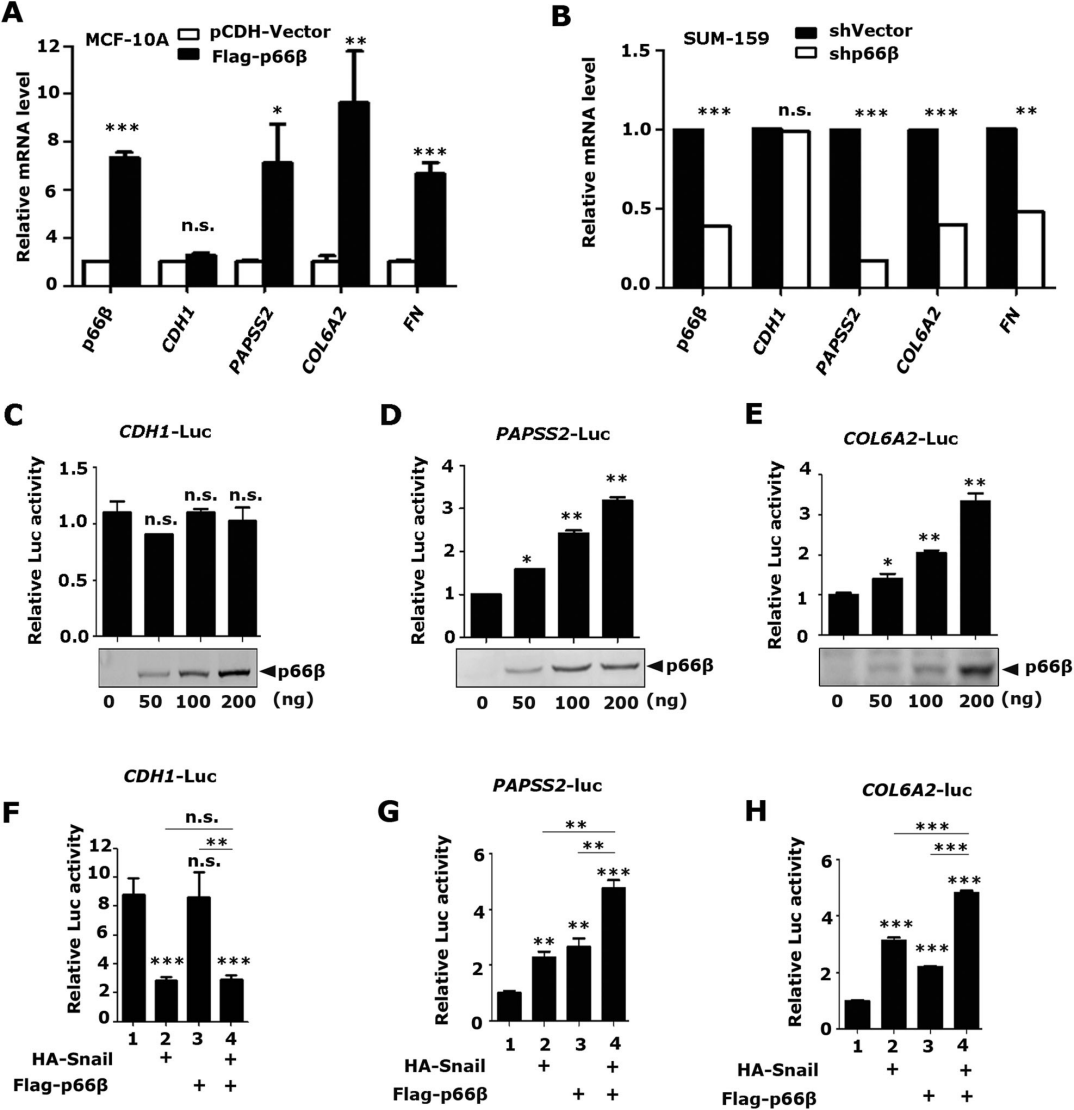
Snail and p66β cooperatively transactivate genes. **A** Stably expressed p66β didn’t induce the mRNA level of *CDH1* but markedly increased the mRNA level of *PAPSS2*, *COL6A2*, and *FN*. The relative mRNA expression of *CDH1*, *PAPSS2*, *COL6A2*, and *FN* was detected by qRT-PCR and normalized by the expression of β-actin. *p < 0.05, **p < 0.01, ***p < 0.001 and n.s.: no significance. **B** Depletion of p66β didn’t affect the mRNA level of *CDH1* but resulted in decreased mRNA levels of *PAPSS2*, *COL6A2*, and *FN*. **p < 0.01, ***p < 0.001, n.s.: no significance. **C**–**E** p66β didn’t induce the *CDH1*-Luc (**C**) promoter activity, but the *PAPSS2*-Luc (**D**), and *COL6A2* -Luc (**E**) promoter activity. The reporters were co-transfected with HA-p66β in HEK-293T cells. Reporter assays were performed and the luciferase activity was normalized to β-galactosidase activity. *p < 0.05, **p < 0.01 and n.s.: no significance. **F**–**H** p66β and Snail cooperatively induced the *PAPSS2* and *COL6A2* promoter activity, not the *CDH1* promoter activity. **p < 0.01, ***p < 0.001 and n.s.: no significance. All data are shown as the mean ± S.D. from three independent experiments by Student’s t-test.

To determine whether p66β participates in Snail-mediated gene transcription, we first examined the best-known Snail target genes such as *CDH1*, *FN*, as well as *PAPSS2*, and *COL6A2* which were induced by Snail in our previous RNA-seq analysis [40]. Neither ectopic expression nor depletion of p66β affected the mRNA level of *CDH1*. However, the expression of p66β apparently increased the mRNA levels of the *PAPSS2, COL6A2*, and *FN* genes (Fig. 4A, B), suggesting that p66β may participate in Snail-mediated gene induction rather than repression.

Next, the *CDH1*-Luc, *PAPSS2*-Luc, or *COL6A2*-Luc reporters, together with plasmids encoding Flag-p66β, were transiently transfected into HEK-293T cells, and the luciferase activity was normalized to β-galactosidase activity. Consistently, p66β showed no effect on the *CDH1-*Luc reporter activity (Fig. 4C), but stimulated *PAPSS2*-Luc and *COL6A2*-Luc reporter activities in a dosage**-**dependent manner (Fig. 4D, E). Moreover, Snail alone inhibited *CDH1* promoter activity, and adding p66β did not apparently affect the inhibitory effect of Snail (Fig. 4F). Conversely, Snail or p66β alone weakly or modestly induced *PAPSS2*-Luc and *COL6A2*-Luc activity, whereas co-expression of Snail and p66β markedly induced *PAPSS2*-Luc and *COL6A2*-Luc activity (Fig. 4G, H), indicating that p66β cooperates with Snail to induce target gene expression.

To explain the paradox that the NuRD transcriptional repression complex component p66β is involved in Snail-mediated transcriptional activation, Flag-p66β, HA-MTA2, and/or HA-Snail were co-expressed in HEK-293T cells and co-IP assays were performed. The co-eluted bands showed that MTA2 and Snail could be pull-downed by Flag-p66β, respectively. The interaction between p66β and MTA2 weakened with the increased expression of HA**-**Snail (Fig. S3), indicating that Snail competes with the MTA2 protein for p66β.

### Snail-induced genes contain G-box elements in their proximal promoter regions

**Fig. 5:**
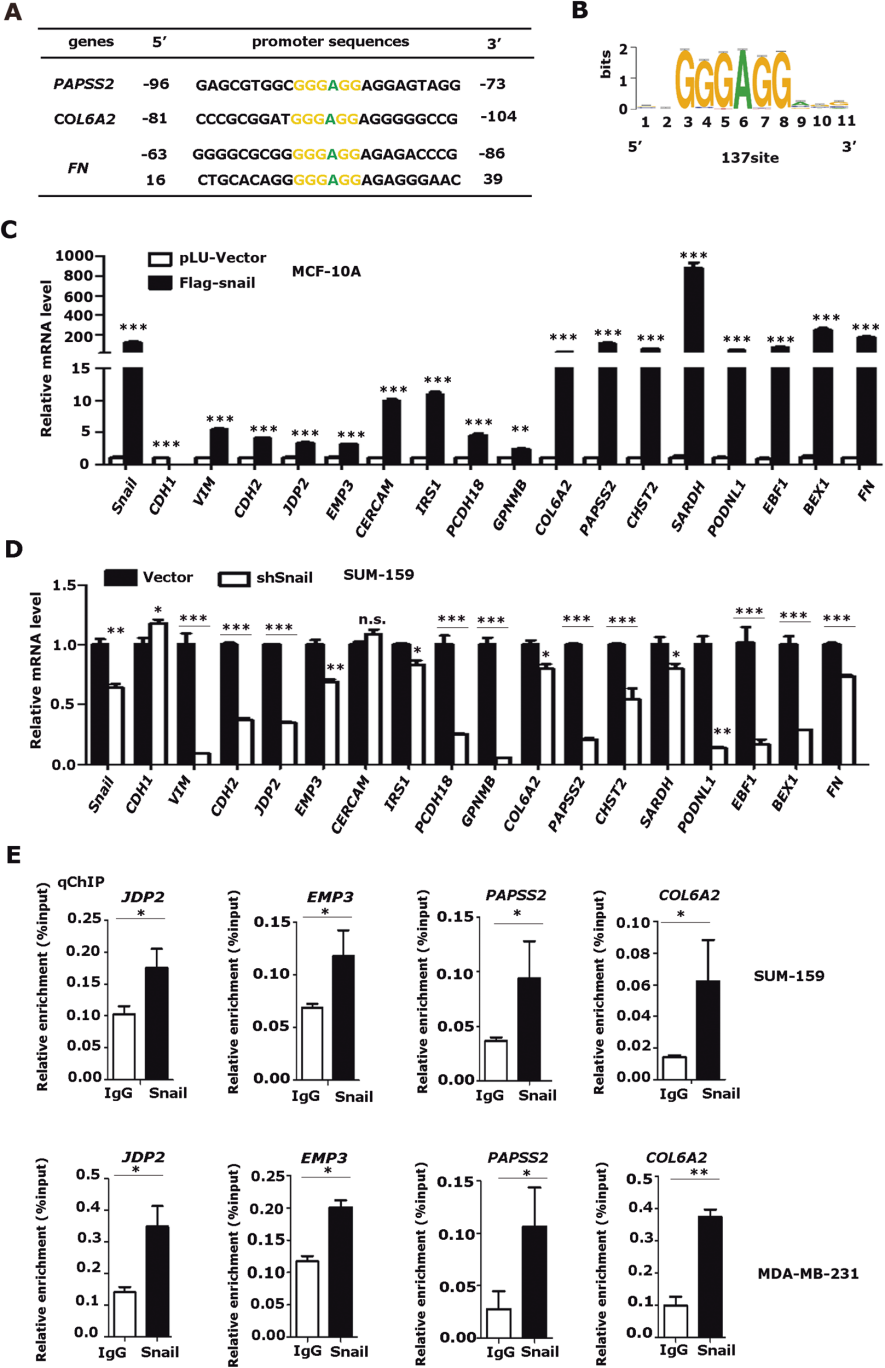
Snail induces transcription of genes containing G-box elements in the proximal promoter regions. **A** The genes induced by Snail contained the 5’-GGGAGG-3’ (G-box) motif in their promoters. **B** Prediction of Snail binding sequences by using RSAT software from a published Snail ChIP-seq data in tumor cells prepared from *MMTV-PyMT* mice and 137 G-box sites, where Snail displayed peak binding activities, were identified. **C** The mRNA levels of genes containing G-boxes were increased in Snail stably expressed MCF-10A. The relative mRNA level of genes was detected by qRT-PCR and normalized by the expression of β-actin. **p < 0.01, ***p < 0.001. **D** The mRNA levels of genes containing G-boxes were reduced in SUM159-shSnail cells. *p < 0.05, **p < 0.01, ***p < 0.001, and n.s.: no significance. **E** Snail bound the proximal promoter regions flanking the G-box motif. The ChIP assays were performed in SUM-159 and MDA-MB-231 cells with a specific antibody against Snail and the enriched DNA fragments were examined by qRT-PCR and normalized to input. *p < 0.05, **p < 0.01. All data are shown as the mean ± S.D. from three independent experiments by Student’s t-test.

By analyzing the promoter sequences of Snail-induced genes, a conserved G-rich cis-element (5′-GGGAGG-3′, designated as G-box herein) was identified in their proximal promoter regions (Fig. 5A). To strengthen this observation, we analyzed the published ChIP-seq data of Snail binding sites in tumor cells prepared from *MMTV-PyMT* mice using RSAT software and identified 137 G-box sites where Snail displayed peak binding activities (Fig. 5B and Fig. S4A, B) [8]. Among the 137 G-box sites, we selected 14 genes conserved in humans and mice and then examined their mRNA levels in human and mouse mammary cells that stably expressed Snail, respectively (Table 1). Indeed, Snail induced the expression of the selected representative genes (Fig. 5C and Fig. S4C). Conversely, depletion of Snail by using specific shRNAs decreased the mRNA levels of these genes (Fig. 5D). Notably, an antibody specific to Snail readily enriched the DNA fragments flanking the G-box elements in the representative promoters of *JDP2*, *EMP3*, *COL6A2*, and *PAPSS2* examined by chromatin immunoprecipitation assays (Fig. 5E). Taken together, these data indicated that Snail can induce the transcription of genes containing G-box elements in their promoters and that the G**-**box element is a potential Snail-responsive element.

**Table 1:**
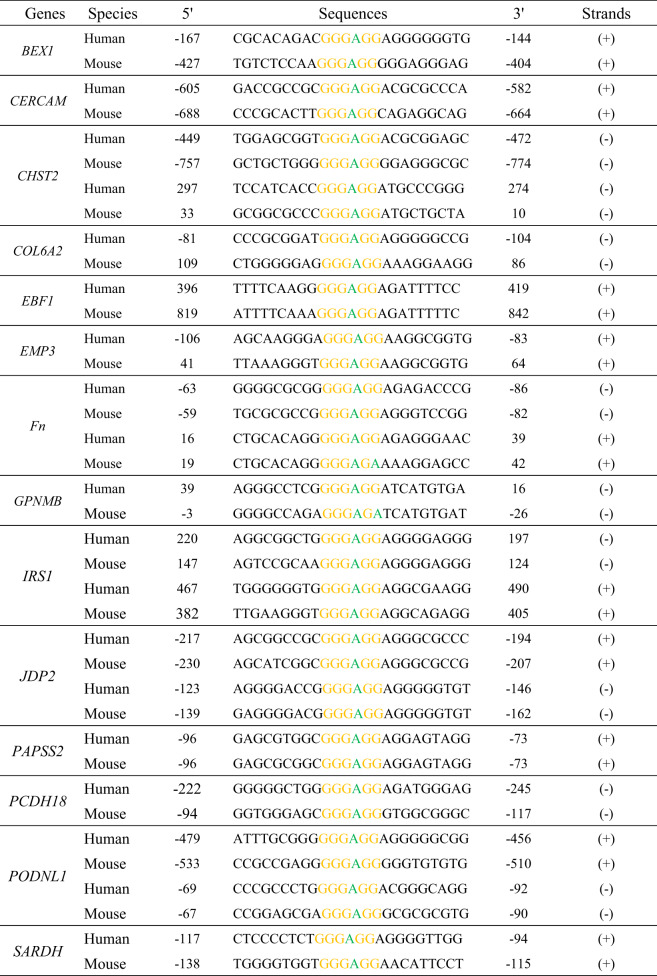
The G-box sequence is highly conserved in human and mouse gene promoters.

, G-box cis-element. (+), sense strand. (-), antisense stand.

### Snail induces transcriptional activity of promoter reporters driven by G-box element

**Fig. 6:**
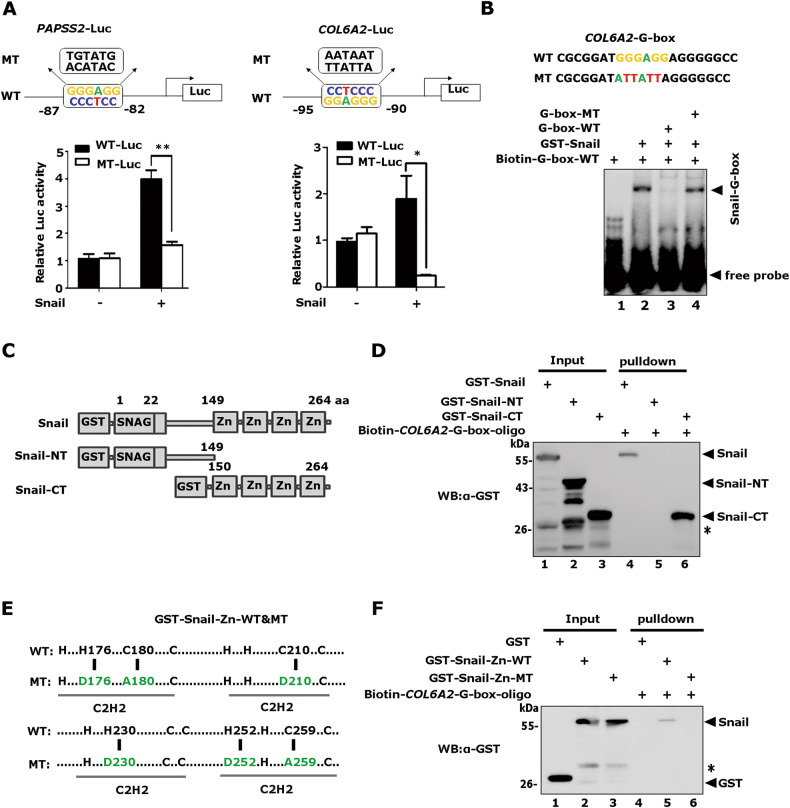
Snail directly binds the G-box elements through its zinc fingers. **A** Snail specifically stimulated the G-box-driven reporter activities. The *COL6A2*-G-box, *PAPSS2*-G-box, and their G-box mutation-driven luciferase reporters were transfected with HA-Snail together in HEK-293T cells and the luciferase activity was normalized to β-galactosidase activity. *p < 0.05,**p < 0.01. The data are shown as the mean ± S.D. from three independent experiments by Student’s t-test. **B** Electrophoretic mobility shift assays (EMSA) were performed by incubating biotinylated *COL6A2*-G-box probes with bacterially expressed GST-Snail protein. Unlabeled wild-type and mutant probes were included as indicated. **C** Schematic diagrams showed full-length and truncated Snail proteins. **D** The biotinylated *COL6A2*-G-box probes and NeutrAvidin beads were incubated with bacterially expressed full-length GST-Snail, Snail-NT (the SNAG domain), or Snail-CT (the zinc-finger region), anti-GST was used to detect the *COL6A2*-G-box probes-bound proteins. *non-specific band. **E** Schematic diagrams showed wild type and zinc finger mutant of Snail proteins. **F** The zinc finger mutant failed to bind *COL6A2*-G-box probes. *non-specific band.

To determine whether Snail directly modulates the activity of the G-box element, we first mutated the G-box of *PAPSS2*-Luc. The two reporters, together with plasmids encoding HA-Snail, were transiently transfected into HEK-293T cells and showed four-fold Snail-induced *PAPSS2*-Luc reporter activity but had no effect on the mutant reporter (Fig. 6A left). Similarly, Snail induced the activity of the *COL6A2*-Luc reporter containing a G-box element, but not a mutant reporter (Fig. 6A right). Moreover, deletion of the SNAG domain of Snail did not obviously affect Snail-mediated induction of promoters with G-boxes (Fig. S5). These data demonstrated that Snail induces the transcriptional activity of promoters containing G-box elements.

Next, we performed electrophoretic mobility shift assays (EMSA) to verify the direct binding between Snail and G-box elements. An up-shifted band was observed after incubation of GST-Snail protein prepared from bacterial cells with biotin-labeled G-box oligonucleotides of *COL6A2* (Fig. 6B, lane 2). The shifted band disappeared with the addition of unlabeled wild-type oligonucleotides but not with unlabeled mutant oligonucleotides (Fig. 6B, lane 3, 4). These data indicated that the Snail protein specifically binds to G-box elements in vitro.

### Snail directly binds G-box elements via its zinc finger motifs

To determine the regions of the Snail protein that can directly bind to the G-box element, full-length GST-Snail, and its truncated mutants were incubated with biotin-labeled G-box oligonucleotides of *COL6A2*. The protein/DNA complex was co-precipitated with NeutrAvidin beads and the co-eluted Snail protein was examined by Western blotting. Both full-length Snail and the truncated C**-**terminal zinc finger mutant (GST-Snail-CT) readily bound to the *COL6A2*-G-box element, while the SNAG domain mutant (GST-Snail-NT) did not (Fig. 6C, D). Moreover, we disrupted the zinc finger structure by simultaneous mutations of H176D, C180A, C210D, H230D, H252D, and C259A, and the mutant protein failed to bind to the *COL6A2*-G-box element (Fig. 6E, F). Collectively, these data demonstrated that Snail directly binds to the G-box element via its zinc finger motifs.

### p66β protein enriches on the promoters flanking G-boxes

**Fig. 7:**
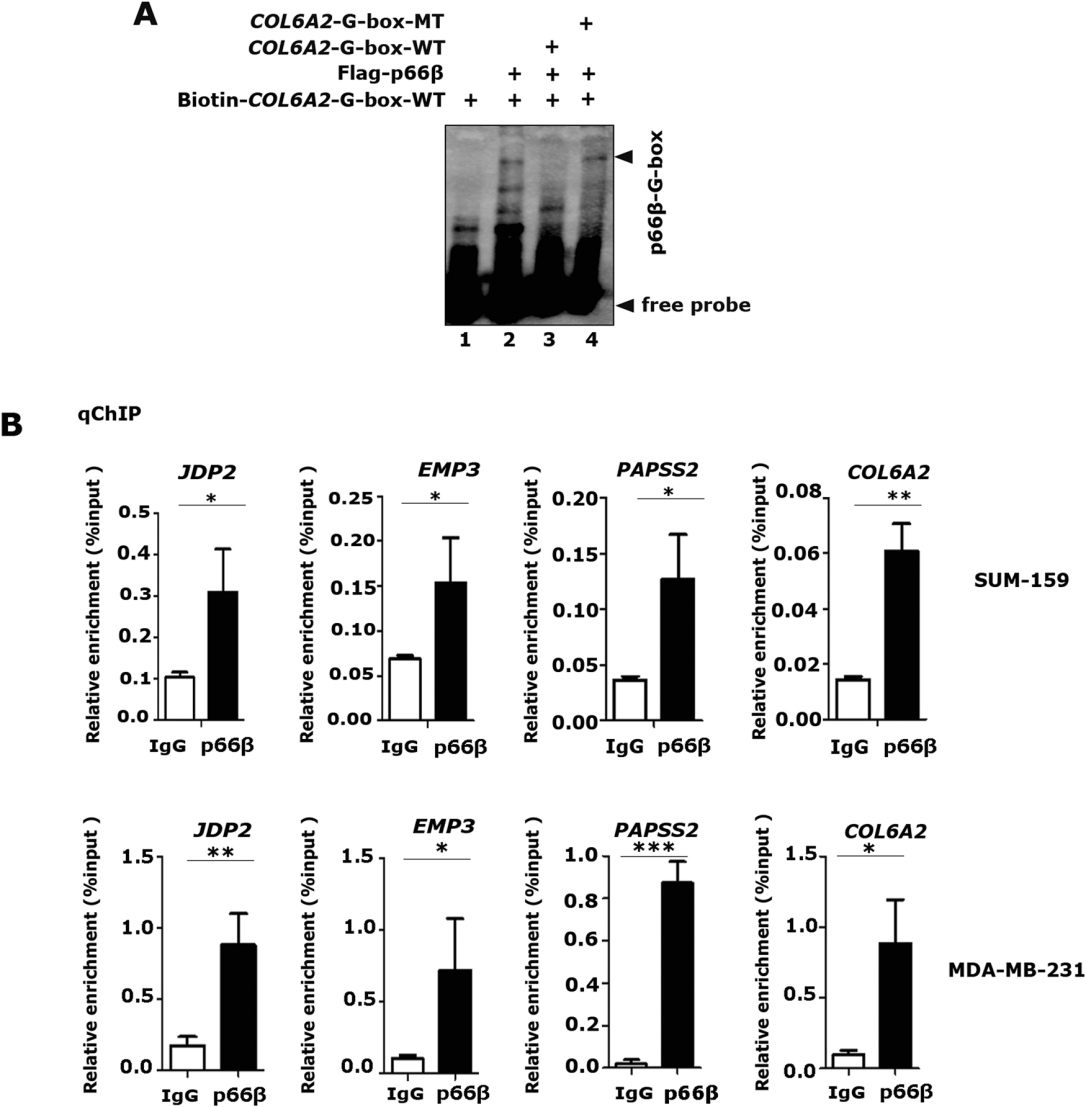
p66β enriches on the promoters flanking G-boxes. **A** EMSA assays were performed by incubating biotinylated *COL6A2*-G-box probes with Flag-p66β protein prepared from HEK-293T-Flag-p66β cells. Unlabeled wild-type and mutant probes were included as indicated. **B** p66β protein bound the promoter region flanking the G-box motif. The ChIP assays were performed in MDA-MB-231 and SUM-159 cells with a specificity antibody against p66β and the enriched DNA fragments were examined by qRT-PCR and normalized to input. *p < 0.05, **p < 0.01, and ***p < 0.001. The data are shown as the mean ± S.D. from three independent experiments by Student’s t-test.

To determine whether p66β can directly bind to the G-box element, EMSA assays were performed by incubating the biotinylated *COL6A2*-G-box with cell extracts prepared from HEK-293T cells expressing wild-type Flag-p66β. Remarkably, adding p66β protein resulted in shifted bands containing the biotinylated oligonucleotide *COL6A2*-G-box elements, which disappeared upon the addition of unlabeled wild-type oligonucleotides, but not the unlabeled mutants (Fig. 7A). ChIP assays using p66β antibodies indicated that p66β enriched endogenous DNA fragments from the promoters of *JDP2*, *EMP3*, *PAPSS2*, and *COL6A2* flanking G-box sites (Fig. 7B). Taken together, these data demonstrated that p66β enriches on the promoter flanking G-boxes.

### p66β protein is indispensable for Snail to bind the G-box elements

**Fig. 8:**
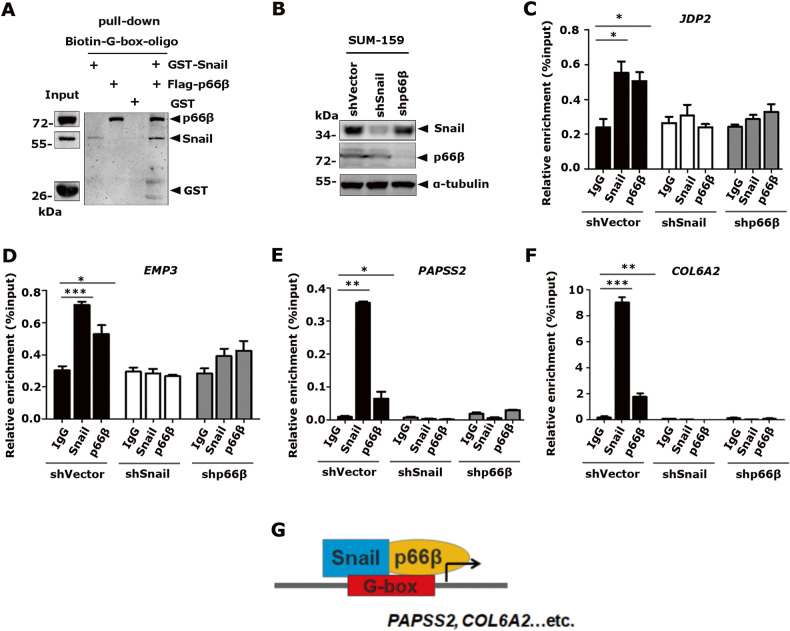
p66β is indispensable for Snail to bind the G-box elements. **A** p66β enhanced the Snail binding to G-box. Biotinylated *COL6A2*-G-box probes and NeutrAvidin beads were incubated with bacterially expressed GST-Snail, and/or Flag-p66β proteins from HEK-293T-p66β cells, Anti-GST, and anti-Flag were used to detect *COL6A2*-G-box probe-bound proteins. **B** Western blotting showing the levels of Snail and p66β proteins in SUM-159 cells transfected with specific shRNAs. **C**–**F** Knocking-down of p66β inhibited the binding affinity of Snail to the proximal promoter regions of *JDP2*, *EMP3*, *PAPSS2*, and *COL6A2* flanking identified G-boxes. ChIP assays in SUM-159 cells expressing shVector, shp66β, or shSnail were performed by using specific antibodies against IgG, Snail, and p66β. The relative enrichment of Snail and p66β on the promoters of *JDP2* (**C**)*, EMP3* (**D**)*, PAPSS2* (**E**), and *COL6A2* (**F**) was examined by qRT-PCR and normalized to the inputs. *p < 0.05, **p < 0.01, and ***p < 0.001. The data are shown as the mean ± S.D. from three independent experiments by Student’s t-test. **G** Model in which Snail transactivates gene expression by recruiting p66β protein.

To examine the role of p66β in Snail-mediated transactivation, we performed DNA pull-down assays to examine the binding of Snail to the G-box elements in the presence of p66β. Surprisingly, p66β alone bound the G-box probes with a relatively high affinity and enhanced Snail binding to the G-box probes (Fig. 8A).

To further examine the binding activity of Snail and p66β at the endogenous target chromatins, we performed ChIP assays in SUM-159 cells bearing specific shRNAs to deplete p66β or Snail expression (Fig. 8B). Consistently, both Snail and p66β were associated with the promoter chromatin flanking the G-box elements of *JDP2*, *EMP3*, *PAPSS2*, and *COL6A2* in SUM-159-shVector cells, whereas depletion of p66β or Snail, respectively, resulted in decreased binding of Snail and p66β to these promoters (Fig. 8C–F). Collectively, these data demonstrated that p66β is required for Snail to efficiently bind G-box elements.

## Discussion

In this study, we demonstrated that Snail cooperates with p66β to transactivate target gene transcription by directly binding G-box elements in their promoters and that the induction of gene expression by Snail does not require the SNAG domain (Fig. 8G). Snail interacts with the CR2 domain of p66β via the zinc fingers. Snail by itself weakly binds to the G-box elements, while p66β can markedly enhance Snail binding affinity to G-box elements, and depletion of p66β results in diminished Snail binding to the endogenous promoters, indicating that p66β is critical for Snail-mediated transactivities. More importantly, ectopic expression of p66β promotes breast cancer cell migration and metastasis, whereas depletion of p66β is detrimental to Snail-mediated cell migration and metastasis. Collectively, these data indicated that Snail cooperates with p66β to induce gene expression by directly binding to the G-box elements in the promoters.

Growing evidence has indicated that Snail functions as a transcriptional activator and directly induces gene expression. Earlier studies found that the Snail transcriptional activation recognition motif is still E-box. The E-boxes in the regulatory region of *ERCC1* and *MMP15* were recognized by Snail [24, 25]. As the research unfolded, it was found that Snail recognition sequences were not limited to the E-box, the new binding sequences were discovered. A novel Snail-responsive motif, 5′-TCACA-3′, in the promoters of *ZEB1*, *MMP9*, and *p15*^*INK4b*^, has been identified and activated by Snail in collaboration with EGR-1 and SP-1 [28, 29]. In this study, we denote, G-box (5′-GGGAGG-3′), a new element recognized by zinc fingers in the C-terminal of Snail and important for transactivation. Interestingly, a fragment of the *Fibronectin1* promoter containing G-box, 5′-GGGGGAGGAGAGGGAACCCCAGGCGCGAGC-3′, was bound by Snail extraction, not the GST tagged Snail [30]. Whether there are similar consensus binding motifs for Snail to upregulate gene expression is worth investigating in the future.

The role of the SNAG domain in Snail-mediated gene induction is controversial. The SNAG domain of Snail is essential for both the suppression of E-cadherin and the activation of ERCC1 in head and neck squamous cell carcinoma cells [24], while in another report, the transactivation of β-catenin-dependent reporter by Snail does not require the SNAG domain but the zinc fingers [41]. In this study, we showed that deletion of SNAG does not affect the transactivation of target genes.

p66β protein was previously identified as a key component of the NuRD complex, which modifies chromatin structure to maintain gene repression [32, 35, 36]. Notably, Grzeskowiak et al. reported that p66β induces Myc expression in KRAS- mutant lung cancer cells, indicating that p66β could function as a transcriptional activator [39]. Our data demonstrated that p66β can directly bind G-box elements and induces G-box-containing promoter activity. In contrast, p66β does not bind E-box elements and consequently, does not affect E-box-driven promoter activity. The Snail/p66β complex and Mi-2/NuRD exist mutually exclusively in cells since Snail competes with the MTA2 protein for p66β.

Both Snail and p66β contain zinc finger motifs. The snail contains four C2H2 types of zinc fingers. The CR2 domain of p66β contains a GATA zinc finger protein. It is known that zinc fingers are not only responsible for specific DNA binding but also function as protein-protein interacting modules [42, 43]. In this study, we demonstrated that Snail binds to the CR2 domain of p66β, which contains a GATA zinc finger, via its C2H2 zinc fingers. We speculate that Snail and p66β may also dimerize through their zinc finger motifs, resulting in the remodeling of their binding specificity and affinity toward G-box elements to activate gene expression. These studies have uncovered a novel mechanism for Snail-mediated transcriptional activation. The Snail/p66β complex binds to G-box elements to induce target gene expression. Biologically, p66β is critical for Snail to promote cell migration and metastasis.

## Materials and methods

### Plasmids

The pLU-GFP-Flag-Snail, pCMV5-HA-Snail, and pCDNA3.1-Flag-Snail plasmids have been described previously [40]. GST-Snail and its truncated plasmids were subcloned into pGEX-4T-1(28-9545-49, GE Healthcare) by PCR between *Bam*H I (R3136M, NEB) and *Eco*R I (R3101M, NEB) restriction enzyme sites. The GST-Snail-ΔZn was subcloned from GST-Snail using point mutation as indicated in Fig. 6E. The pCDNA-Flag-p66β plasmid was a kind gift from Dr. Renkawitz. Deletion mutants of Flag-p66β and pCDH-Flag-p66β plasmids were subcloned into the pCMV4-Flag-Vector (E7158, Sigma-Aldrich) and pCDH-CMV-MCS-EF1-Puro-Vector (CD510B-1, System Biosciences), respectively. The HA-MTA2 plasmid was subcloned into pCMV5-HA-Vector by PCR between *Bam*H I and *Eco*R I restriction enzyme sites. The pLKO.1-shp66β plasmid was purchased from GE Healthcare, and the short hairpin RNA sequence was 5′-GCTCCATGCTTTCAAACTTT-3′. The oligo of the short hairpin RNA sequence (5′-CCAAGGATCTCCAGGCTCGAA-3′) was inserted into pLKO.1-tet-on-Vector to construct the shSnail inducible plasmids. The promoter DNA sequences of human *PAPSS2* (−96 to +1 bp) and *COL6A2* (−119 to +89 bp) were subcloned into a pGL3-Basic-Vector to create the corresponding luciferase reporters.

### Cell culture, transfection, and lenti-virus infection

MCF-7, T47D, SK-BR-3, MDA-MB-231, SUM-159, 4T1, and HEK-293T cells were maintained in Dulbecco’s modified Eagle’s medium (DMEM) supplemented with 10% (v/v) fetal bovine serum and penicillin (50 U/ml)/streptomycin (50 µg/ml) at 37 °C under 5% CO_2_ in a humidified chamber. MCF-10A cells were maintained in DMEM/F12 supplemented with 5% horse serum, EGF (20 ng/ml), insulin (10 µg/ml), hydrocortisone (0.5 µg/ml), cholera toxin (100 ng/ml), penicillin (50 U/ml), and streptomycin (50 µg/ml) at 37 °C under 5% CO_2_ in a humidified chamber. All cell lines were obtained from American Type Culture Collection (ATCC) (https://www.atcc.org/) and tested negative for mycoplasma contamination.

Transient transfections were carried out using PEI reagent (23966-2, Polysciences) according to the manufacturer’s instructions. For lentiviral infection, viruses were packaged into HEK-293T cells, and injected into MCF-10A, MDA-MB-231, SUM-159, and 4T1 cells.

### Affinity purification of Snail-interacting protein complex

To purify Snail-associated proteins, pcDNA3.1-Flag-Snail plasmids were stably expressed in HEK-293T cells. A total of 5 × 10^9^ cells were lysed in buffer A (20 mM Tris-HCl (pH 8.0), 150 mM NaCl, 2.5 mM EDTA, 0.5%NP-40, 0.2 mM PMSF, and 0.5 mM dithiothreitol (DTT)). Cell lysates were precleared with the protein A/G agarose (sc-2003, Santa Cruz) for 2 h and then incubated with the anti-Flag M2 affinity gels (F2426, Sigma-Aldrich) at 0.5 ml of beads per 100 mg of cell lysate for 2 h to overnight with rotation. The anti-Flag M2 gels were washed four times with buffer BC500 containing 20 mM Tris-HCl (pH 7.8), 500 mM KCl, 0.2 mM EDTA, 10% glycerol, 10 mM β-mercaptoethanol, 0.2% NP-40, 0.2 mM PMSF, and protease inhibitor cocktail. The protein complexes were eluted with the 3×Flag peptides (F4799, Sigma-Aldrich) at 0.4 mg/ml in buffer BC100 containing 20 mM Tris-HCl (pH 7.8), 50 mM KCl, 0.2 mM EDTA, 10% glycerol, 10 mM β-mercaptoethanol, 0.2 mM PMSF, and protease inhibitor cocktail. The eluted proteins were resolved on 4 to 12% SDS-PAGE gels for Western blotting and colloidal staining analyses. The proteins were excised from the gels and identified by standard mass spectrometry.

### Co-immunoprecipitation (Co-IP), Western blotting (WB), immunofluorescence (IF), and antibodies

For Co-IP assays, HEK-293T cells were transfected as indicated: 36 h later, cells were lysed in IP buffer (1% Triton X-100, 150 mM NaCl, and 50 mM Tris/HCl (pH 7.5), 1 mM PMSF, and protease inhibitor cocktail) and incubated with anti-Flag M2 affinity gels for 4 h at 4 °C. For reverse IP, HEK-293T cells were lysed and incubated with anti-HA antibody and protein A/G agarose for 12 h at 4 °C. Agarose was washed five times with IP assay buffer, boiled in SDS sample buffer, and subjected to Western blot analysis. Western blotting and immunofluorescence (IF) were performed as previously [40].

The following antibodies were used: mouse anti-Flag (F3165, Sigma-Aldrich), rabbit anti-Flag (F7425, Sigma-Aldrich), rabbit anti-GST (2622, Cell Signaling Technology), mouse anti-β-actin (66009-1-Ig, Proteintech), rabbit anti-Snail (3879, Cell Signaling Technology), mouse anti-Snail (sc-271977, Santa Cruz), rabbit anti-p66β (ab76925, Abcam), anti-biotin, HRP-linked (7075, Cell Signaling Technology), and rabbit anti-IgG (2729, Cell Signaling Technology), mouse anti-α-tubulin (66031-1-Ig, Proteintech), rabbit anti-Fibronectin1 (15613-1-AP, Proteintech), rabbit anti-E-cadherin (20874-1-AP, Proteintech), rabbit anti-Vimentin (10366-1-AP, Proteintech).

### Luciferase reporter assay, quantitative real-time PCR (qRT-PCR), and chromatin immunoprecipitation (ChIP) assay

Luciferase reporter assays and quantitative real-time PCR (qRT-PCR) were performed as described [40].

ChIP assays were performed using 5 × 10^6^ cells and antibodies against Snail (3879, Cell Signaling Technology) and p66β (ab76925, Abcam). Cells were cross-linked with 1% formaldehyde for 10 min at room temperature, followed by quenching with 125 mM glycine (50046, Sigma-Aldrich). After washing with PBS, cells were scraped and centrifuged. The cell pellets were sonicated in lysis buffer (150 mM NaCl, 50 mM Tris-HCl (pH 7.5), 5 mM EDTA, NP-40 (0.5% vol/vol), Triton X-100 (1.0% vol/vol), 1 mM PMSF, and protease inhibitor cocktail) using a sonicator (Bioruptor Plus, Diagenode) and the fragment length (200–500 bp) was determined by agarose gel electrophoresis. After incubation with beads and antibodies, the ChIP DNA was eluted with elution buffer (50 mM Tris-HCl, pH 8.0, 10 mM EDTA, 1.0% SDS) and incubated with RNAase (R6513, Sigma-Aldrich) at 37 °C for 1 h and Proteinase K (p2308, Sigma-Aldrich) at 55 °C for 2 h. The final ChIP products were purified using an EZNA Cycle-Pure kit (D6492-02, Omega) and analyzed by qRT-PCR using SYBR Green on a Roche system (LightCycler 480II). The comparative cycle threshold method was used to determine the enrichment relative to the input level. The assays were replicated three times. The primer sets used for the qRT-PCR and ChIP assays are listed in Table 2.

**Table 2:** Primer list.

Primers for qPCR		
Name	Forward sequence	Reverse sequence
β-actin-226-F/417-R	5′-TCACCAACTGGGACGACAT-3′	5′-CACAGCCTGGATAGCAACG-3′
Snail-F-58-F/193-R	5′-GCGAGCTGCAGGACTCTAAT-3′	5′-GGACAGAGTCCCAGATGAGC-3′
CDH1-1502-F/1592-R	5′-TGGGCCAGGAAATCACATCCTACA-3′	5′-TTGGCAGTGTCTCTCCAAATCCGA-3′
CDH2-449-F/-649-R	5′-ACAGTGGCCACCTACAAAGG-3′	5′-CCGAGATGGGGTTGATAATG-3′
Fibronectin1-6507-F/6838-R	5′-GAATGTAGGACAAGAAGCTC-3′	5′-CATCTCCAACGGCATAATGG-3′
Vimentin-1045-F/1207-R	5′-GAGAACTTTGCCGTTGAAGC-3′	5′-GCTTCCTGTAGGTGGCAATC-3′
JDP2-191-F/439-R	5′-TGGAGGTGAAACTGGGCAAGA-3′	5′-GGTGTCGGTTCAGCATCAGG-3′
EMP3-22-F/171-R	5′-GTCTCAGCCCTTCACATCCT-3′	5′-GACATTACTGCAGGCCCATG-3′
CERCAM-635-F/-818-R	5′-TGGTCCACTCCACCTTCCTT-3′	5′-GGCACATTCATGTACCCATAACG-3′
IRS1-565-F/729-R	5′-AGCAAGACCATCAGCTTCGT-3′	5′-AGAGTCATCCACCTGCATCC-3′
PCDH18-1550-F/729-R	5′-ATGTAACCATTGACCCAT-3′	5′-ATTGCCCTTATTCTTGTG-3′
GPNMB-1476-F/1644-R	5′-GCCTTTAAGGATGGCAAACA-3′	5′-TGCACGGTTGAGAAAGACAC-3′
PODNl1-169-F/334-R	5′-GACTGTGATGGCTTGGACCT-3′	5′-CTTCGGAGGAGATGAGGTTG-3′
PAPSS2-1488-F/1785-R	5′-CAGTTGCGCAATCCTGTCCACAAT-3′	5′-AGAAATTGGCACCCGCAATCATCC-3′
CHST2-1322-F/1485-R	5′-TTTTGTGGGACTGTTGGTGA-3′	5′-CACCTGTTTGATCTGCTGGA-3′
COL6A2-1872-F/2141-R	5′-GAGCATTGGGTACACCAACTTCA-3′	5′-AGGCGGTCGTAGGCAAACTT-3′
BEX1-109-F/323-R	5′-CCTTTGGATGCTGGTGAAT-3′	5′-ACTGCCCGCAGACTATGAC-3′
SARDH-678-F/1064-R	5′-CCGAGGAGCACAGGTCATT-3′	5′-TCCCAGTCCAGGTCAAAGAG-3′
EBF1-187-F/342-R	5′-CGGAAATCCAACTTCTTCCA-3′	5′-AAGCTGAAGCCGGTAGTGAA-3′
p66β-257-F/577-R	5′-ATGGAGACAACAGGACTGCTGGAA-3′	5′-TGGGCAGGAGATGGCTGAACAATA-3′
mβ-actin-233-F/471-R	5′-ACTGGGACGACATGGAGAAG-3′	5′-GTCTCCGGAGTCCATCACAA-3′
mSnail-84-F/291-R	5′-TTTACCTTCCAGCAGCCCTA-3′	5′-CCCACTGTCCTCATCTGACA-3′
mPapss2-1181-F/1427-R	5′-TTACGCCTCTGGAGCTCAAA-3′	5′-ACCCTTTCCTCCAGTACAGC-3′
mJdp2-186-F/364-R	5′-ACCCGTGAAGAGTGAGCTAG-3′	5′-TCAGCTCCTCTATCTGCGTC-3′
mCol6a2-1853-F/2018-R	5′-GATGCTGCGACTGTGAGAAG-3′	5′-GTGCCTGTTTCTGACTTGGG-3′
mChst2-1259-F/1498-R	5′-ACTTGGTGGTGAGGTACGAG-3′	5′-AGCAAAACTCCTCCACCTGT-3′
mCercam-866-F/1072-R	5′-TGGAAGCACTAGTGGATGGG-3′	5′-TGTTGAGTGTCCTGCCATCT-3′
mIrs1-465-F/706-R	5′-CAAGGAGGTCTGGCAGGTTA-3′	5′-CCACTTGCATCCAGAACTCG-3′
mBex1-87-F/306-R	5′-TACCATCAGAAGGGAGCCAG-3′	5′-CCTTTCCCTCAGCTTCTCCA-3′
mEbf1-1351-F/1558-R	5′-GCATCACAAGCCACCAATCA-3′	5′-GCACAATGGCATAGGGTGAG-3′
mPcdh18-1165-F/1410-R	5′-TCTGGGCTGAATGGGGAAAT-3′	5′-ATATCGGCTCCTCTGGAAGC-3′
mPodnl1-328-F/554-R	5′-GATGAGGCCTTTGAGTCCCT-3′	5′-TGGAAGGTGTTAGGTGGCAA-3′
mGpnmb-1281-F/1516-R	5′-AGCCTGTACGATCATCTCCG-3′	5′-TCAGGACACCATTCACTGCT-3′
mEmp3-101-F/250-R	5′-AAGAGTCCCTGAACCTGTGG-3′	5′-AGAGGATGAAGGACAGGCAG-3′
mSardh-1490-F/1658-R	5′-GCTGTGTGTTTCAAGAGCGA-3′	5′-GGTGGGAAGTCGAAGGTGTA-3′
**Primers for ChIP**		
Name	Forward sequence	Reverse sequence
PAPSS2-qChIP-F/R	5′-GTGGCGGGAGGAGGAGTAG	5′-CTCCCGGGAAGGAGGTATAA-3′
EMP3-qChIP-F/R	5′-AGCTGGGATTACAGGTGTGC	5′-ACGGAGTCTCAAGAGGTTGG-3′
JDP2-qChIP-F/R	5′-CCTCTCGCGTCTCTCTCCTA	5′-CACACCCACTGACCTTAACC-3′
COL6A2-qChIP-F/R	5′-CCACCCTCTCCACCTCCT	5′-TAAGCAGCAGGGAGGGAAC-3′
**Oligonucleotides**	Forward sequence	Reverse sequence
COL6A2-G-box-WT	5′-GGCCCCCTCCTCCCATCCGCG-3′	5′-CGCGGATGGGAGGAGGGGGCC-3′
COL6A2-G-box-MT	5′-GGCCCCCTAATAATATCCGCG-3′	5′-GCGGATATTATTAGGGGGCC-3′

### ChIP-Seq analysis, peak calling, and binding site identification

ChIP-seq raw data were downloaded from the GSE611987 [8]. Reads were mapped to the mouse reference genome mm10 using bowtie (http://bowtie.cbcb.umd.edu) with the parameters -e 70 -k 2 -m 2 -n 2 -best -concise [44]. The seed length (-l) was set to the read length for each data set. Aligned reads were processed using MACS 1.4.2 (http://github.com/taoliu/MACS) with the parameters -w -S -space=50 –keep -dup=auto -p 1e-9 [45]. Snail-binding motifs were identified using the RSAT oligo-analysis tool based on the enriched peak regions (http://copan.cifn.unam.mx/Computational_Biology/yeast-tools) [46].

### Protein purification, electrophoretic mobility shift assay (EMSA), and biotin-DNA pull-down assays

GST-Snail and its truncated proteins were expressed in *E. coli*. BL21 cells (CD601-02, TransGen). The proteins were extracted with GST lysis buffer (25 mM Tris-HCl pH 8.0, 150 mM NaCl, 10% glycerol, 0.1 mg/ml lysosome,1 mM PMSF, and protease inhibitor cocktail) and purified on Glutathione Sepharose (17-0756-01, GE Healthcare). Flag-p66β plasmids were transfected into HEK-293T cells for 48 h, cell lysates were incubated with anti-Flag M2 affinity gels for 4 h at 4 °C, 3×Flag-peptide (0.4 mg/ml) were used to exchange for the Flag-p66β protein.

Snail DNA-binding activity in vitro was examined by EMSA using the LightShift chemiluminescent EMSA Kit (20148, Pierce) following the manufacturer’s protocols. Probes for gel shift assays were synthesized, biotinylated, and annealed in 10× Curt Smart Buffer (NEB). Each EMSA reaction was performed in a final volume of 20 μl. GST fusion proteins (12 μg) were mixed with 600 fmol of probes, 10× binding buffer (100 mM Tris, 500 mM KCl, 10 mM DTT; pH 7.5) (2 μl), 50% glycerol (1 μl), 100 mM MgCl_2_ (1 μl), 1 mg/ml poly (dI.dC) (1 μl), 1% NP-40 (1 μl)), and the presence or absence of unlabeled probes. Electrophoresis was performed on a 6% non-denaturing polyacrylamide gel in 0.5× TBE buffer at 100 V and 4 °C for 2 h, followed by electrophoretic transfer onto a nylon membrane (77016, Pierce). The membrane was then cross-linked with a CL-1000 ultraviolet crosslinker (UVP) and subjected to detection using the Chemiluminescent detection module provided in the kit, following the manufacturer’s protocol. The sequences of the biotinylated probes are listed in Table 2.

Potential Snail-DNA complex-binding abilities were verified using biotin-DNA pull-down assays. GST-Snail and /or Flag-p66β prepared from HEK-293T-p66β cells were incubated with biotinylated oligos of *COL6A2*-G-box and NeutrAvidin agarose resins (29202, Thermo Scientific) for 8 h at 4 °C. The beads were washed five times with IP assay buffer, boiled in SDS sample buffer, and subjected to Western blotting.

### Transwell, metastasis assays, and hematoxylin and eosin (HE) staining

Transwell assays were performed to assess the cell migration capability. To evaluate migration, MCF-10A cells were harvested after serum-free starvation for 6 h and then resuspended in the plain DMEM/F12 medium. A total of 5 × 10^4^ cells were applied into the upper 8-μm pore transwell filters (3495, Corning). Complete DMEM/F12 medium was added to the bottom chamber as attractants. After incubation for 20 h, cells were fixed with 4% paraformaldehyde and stained with coomassie brilliant blue. The non-migrated cells on the top of the chamber were removed gently with cotton swabs, and the migrated cells at the bottom of the filter were quantified by counting five (scale bar, 200 μm) randomly chosen fields using an inverted phase contrast microscope in each experiment. The Experiments were repeated in triplicates. The procedure of migration assays in SUM-159 and MDA-MB-231 cells was essentially the same as that in MCF-10A cells, but with 3 × 10^4^ cells and complete DMEM media as attractants instead. The migrated cells at the bottom of the filter were quantified by counting nine randomly chosen fields (scale bar, 100 μm) using an inverted phase contrast microscope in each experiment. All animal experiments were performed under relevant guidelines and regulations and were approved by the Institutional Animal Care and Use Committee of Shanghai (IACUC: GBT 35892-2018).

Eight-week-old of female BALB/c nude mice were ordered from SLAC Laboratory Co. Ltd (Shanghai, China) and were assigned to experimental groups using simple randomization. 5 × 10^5^ MDA-MB-231 cells (shVector and shp66β) labeled with luciferase were resuspended in 100 μl PBS buffer and were injected into the tail vein of mice (*n* = 10 for each group). Metastatic tumors were examined by an experimenter blinded to injection condition and experimental cohort using the Xenogen IVIS Imaging System biweekly after the mice were deeply anesthetized by ether. Mice were also deeply anesthetized by ether before decapitation, followed by lung enucleation. Of these, one mouse injected with shVector cells died because of overdue stimulation, resulting in 19 mice for analysis.

For hematoxylin and eosin (HE) staining, murine lung tissues were excised, washed in PBS, and fixed in formalin for 48 h. After fixation, the samples were dehydrated in a graded ethanol series, followed by xylene. Samples were then embedded in paraffin, sectioned onto slides (5 mm thick), and allowed to dry. The slides were deparaffinized using a standard procedure (xylene × 2 washes, 100% ethanol × 2 washes, 95% ethanol, 70% ethanol, and 50% ethanol) and stained with hematoxylin and eosin (HE) (HT110132, Sigma-Aldrich).

### Statistical analysis

Data were shown as mean ± SD and were analyzed by the independent Student’s t-test from three independent experiments. The postoperative survival of patients with triple-negative (ER-/PR-/HER2-) breast tumors was analyzed by the Kaplan–Meier estimator (https://kmplot.com/analysis/) and tested by the log-rank. Statistical significance was set at *p* < 0.05.

## Supplementary information

Supplemental figure legends

Supplemental Figure1

Supplemental Figure2

Supplemental Figure3

Supplemental Figure4

Supplemental Figure5

aj-checklist
