# Supplementary material for: GATA zinc finger protein p66β promotes breast cancer cell migration by acting as a co-activator of Snail
Source: Cell Death Dis. 2023 Jun 28;14(6):382. doi: 10.1038/s41419-023-05887-w (PMC10307831; doi:10.1038/s41419-023-05887-w)
Supplement: Supplementary file 1 — Supplemental figure legends [file 41419_2023_5887_MOESM1_ESM.docx]

**Supplementary information**

**Figure S1. The CR2 domain of p66β is responsible for the migration.**

A Expression of p66β, Snail, and EMT markers was determined by Western blotting on non-tumorigenic mammary epithelial (MCF-10A), luminal (MCF-7, T47D), HER2-enriched (SK-BR-3), and triple-negative cell lines (SUM-159 and MDA-MB-231).

B-D Expression of EMT markers was determined by Western blotting on p66β over-expressed (MCF-10A) and knocked down (SUM-159 and MDA-MB-231) cells.

E Western blotting showing the levels of p66β and its truncation proteins in MDA-MB-231 cells transfected with over-expressed plasmids.

F, G The CR2 domain of p66β is responsible for the migration in MDA-MB-231 cells by transwell assays. Images represent one microscopic field in each group (F). Scale bar: 100 μm. Quantification of migrated cells is shown (G) (n=9 fields were randomly chosen and counted for statistical analysis). **p*< 0.05, ***p*< 0.01, n.s.：no significance.

**Figure S2. Snail is required for p66β-mediated cell migration.**

A Western blot analysis of Snail and p66β protein levels in shSnail inducible p66β or Vector stably expressed MDA-MB-231 cells with or without doxycycline (Dox).

B Knockdown of Snail reduced the cell migration mediated by p66β. Scale bar: 100 μm.

C Statistical analysis of the migrating cells from the transwell assays was shown in the bar graphs. (n=9 fields were randomly chosen and counted for statistical analysis). The data are shown as the mean ± S.D. by Student’s t-test. ***p* <0.01.

**Figure S3. MTA2 completes binding site with Snail for p66β.**

Flag-p66β plasmids were transfected with HA-MTA2 and/or HA-Snail plasmids in dose into HEK-293T cells. After 48h of transfection, Co-IP assays were performed using anti-Flag M2 gels, the co-eluted bands showed that MTA2 and Snail could be pull-downed by Flag-p66β, respectively. The interaction between p66β and MTA2 weakened with the increased expression of HA-Snail.

**Figure S4. The summit distribution of Snail ChIP-seq peaks in mice genomic DNA.**

A Among the 19006 peaks Snail enriched, 12421 peaks (65%) were located in the promoter regions.

B Snail binding profile at the promoter loci of the representative gene.

C The mRNA levels of genes containing G-boxes were increased in Snail stably expressed mouse mammary cancer cells 4T1. The relative mRNA level of genes was detected by qRT-PCR and normalized by the expression of β-actin. **p* < 0.05, ***p* < 0.01, and ****p*< 0.001. The data are shown as the mean±S.D. from three independent experiments by Student’s t-test.

**Figure S5. The SNAG domain is not required for the trans-activation of Snail.**

C*OL6A2*-Luc and *PAPSS2*-Luc reporters were transfected with HA-Snail or HA-Snail-ΔSNAG in HEK-293T cells. Reporter assays were performed and luciferase activity was normalized to β-galactosidase activity. n.s.：no significance. The data are shown as the mean±S.D. from three independent experiments by Student’s t-test.
