# Supplementary figures and images for: GATA zinc finger protein p66β promotes breast cancer cell migration by acting as a co-activator of Snail
Source: Cell Death Dis. 2023 Jun 28;14(6):382. doi: 10.1038/s41419-023-05887-w (PMC10307831; doi:10.1038/s41419-023-05887-w)

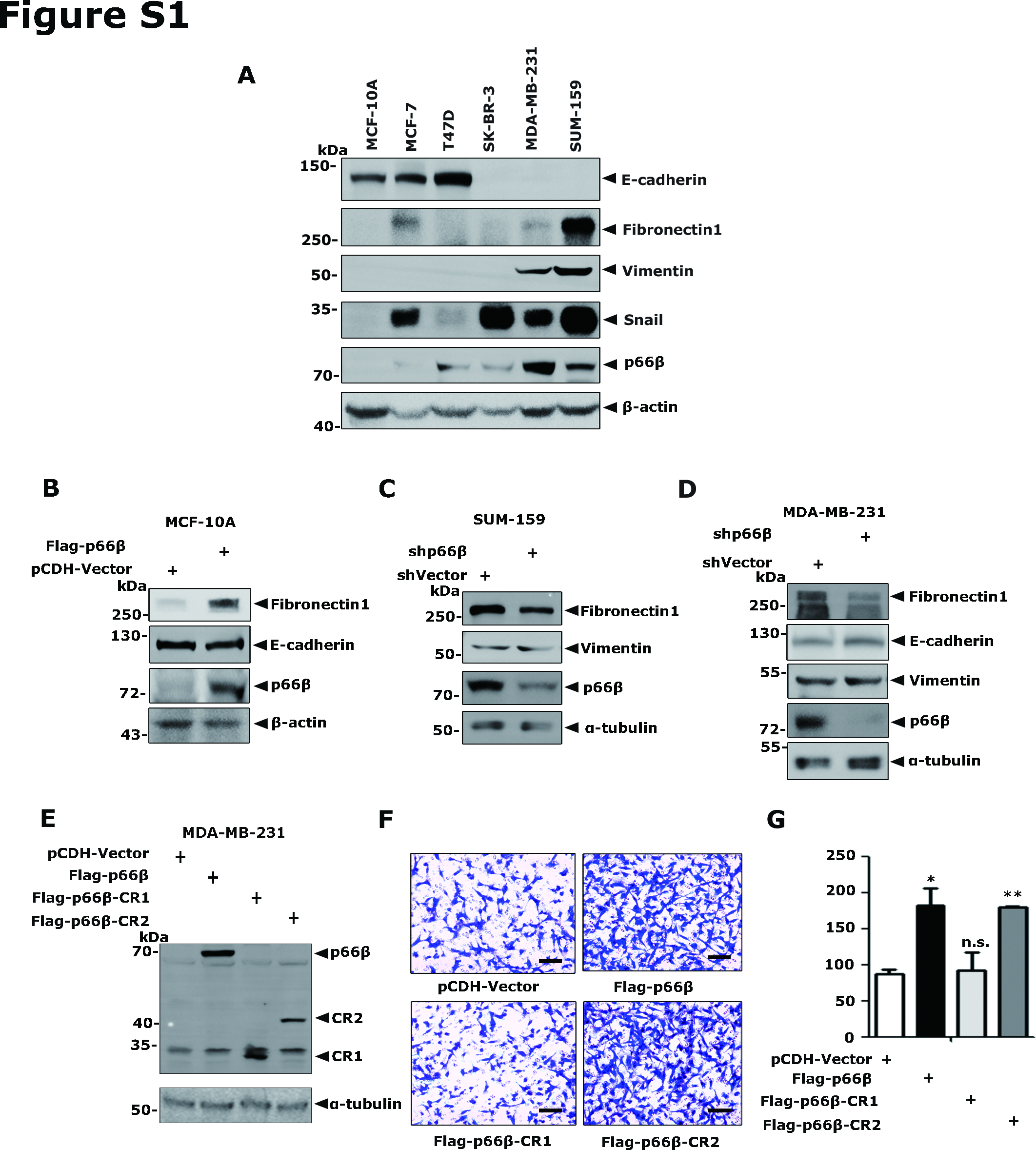

Supplement: Supplementary file 2 — Supplemental Figure1 [file 41419_2023_5887_MOESM2_ESM.tif]

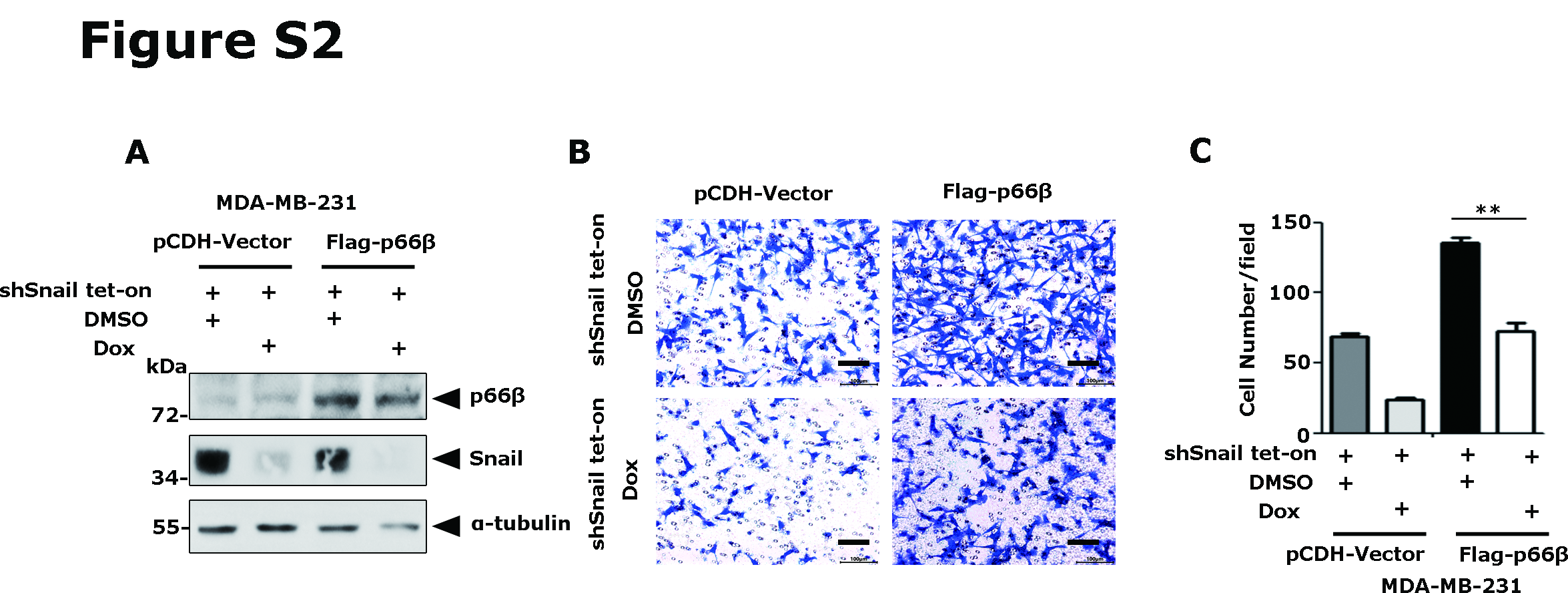

Supplement: Supplementary file 3 — Supplemental Figure2 [file 41419_2023_5887_MOESM3_ESM.tif]

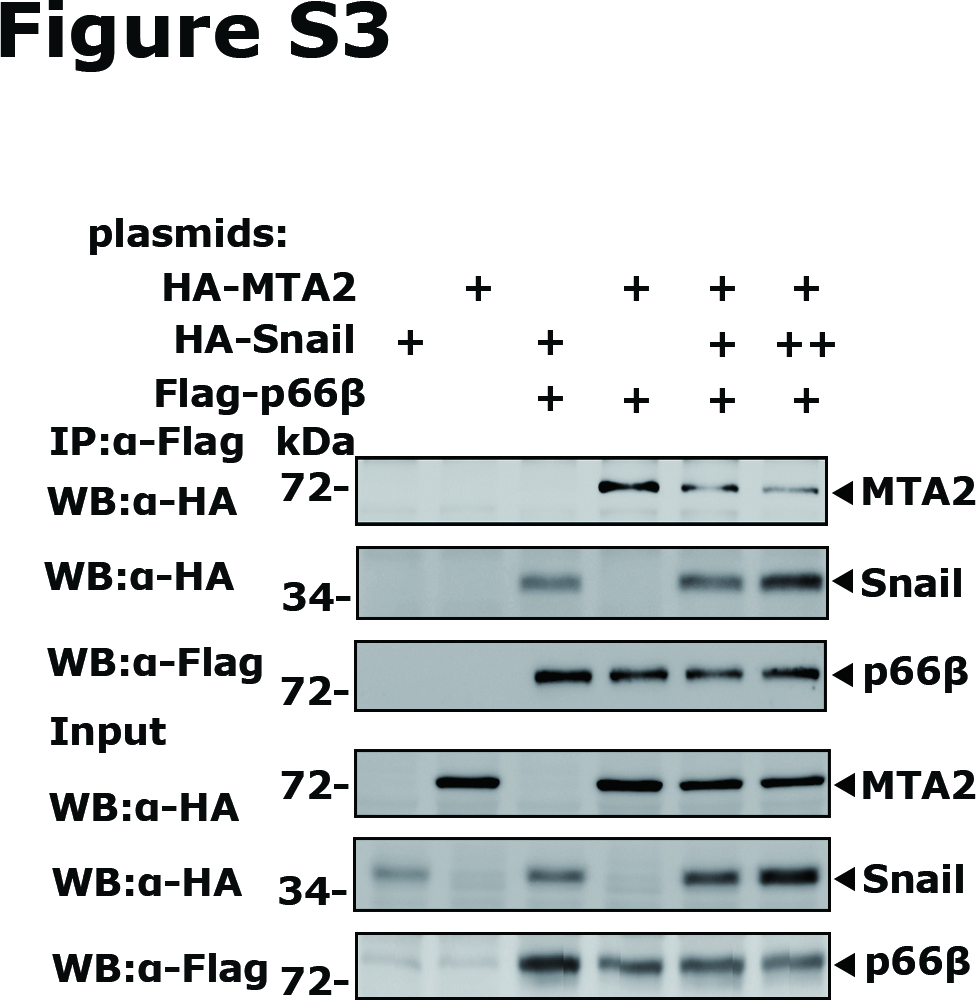

Supplement: Supplementary file 4 — Supplemental Figure3 [file 41419_2023_5887_MOESM4_ESM.tif]

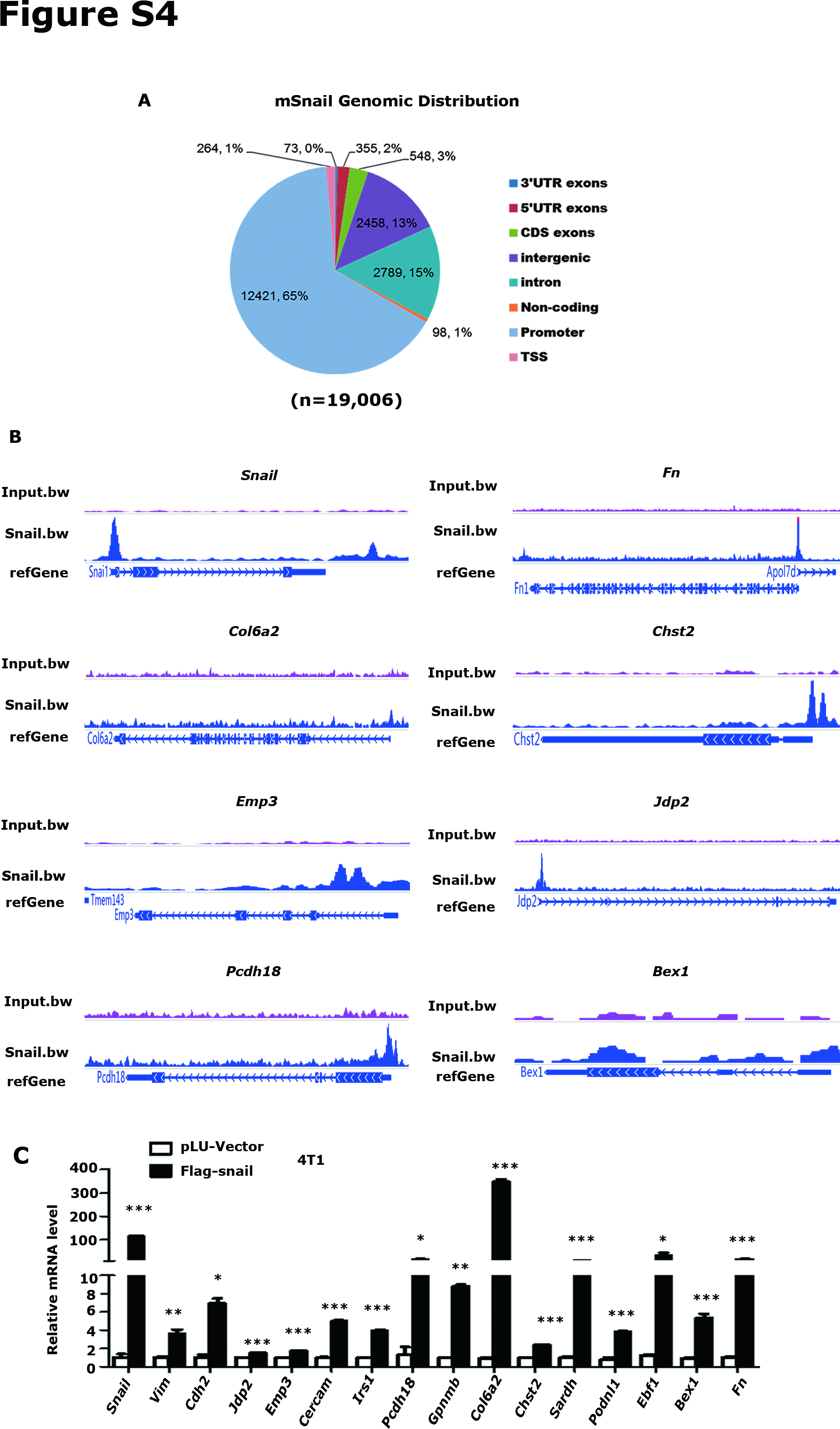

Supplement: Supplementary file 5 — Supplemental Figure4 [file 41419_2023_5887_MOESM5_ESM.tif]

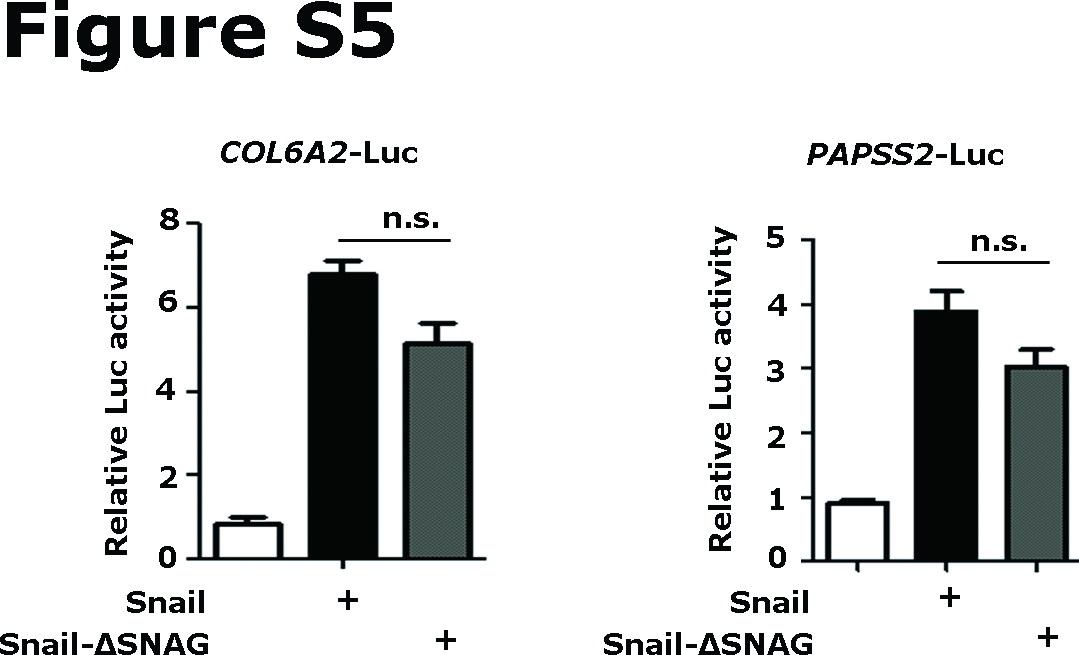

Supplement: Supplementary file 6 — Supplemental Figure5 [file 41419_2023_5887_MOESM6_ESM.tif]
